# Does lean muddy the quality improvement waters? A qualitative study of how a hospital management team understands lean in the context of quality improvement

**DOI:** 10.1186/s12913-016-1838-z

**Published:** 2016-10-19

**Authors:** Carl Savage, Louise Parke, Mia von Knorring, Pamela Mazzocato

**Affiliations:** Medical Management Centre, Department for Learning, Informatics, Ethics and Management, Karolinska Institutet, Tomtebodavägen 18A, SE-17177 Stockholm, Sweden

**Keywords:** Lean, Quality improvement, Pseudoinnovation, Hospital, Management team, Qualitative study

## Abstract

**Background:**

Health care has experimented with many different quality improvement (QI) approaches with greater variation in name than content. This has been dubbed pseudoinnovation. However, it could also be that the subtleties and differences are not clearly understood. To explore this further, the purpose of this study was to explore how hospital managers perceive lean in the context of QI.

**Methods:**

We used a qualitative study design with semi-structured interviews to explore twelve top managers’ perceptions of the relationship between lean and quality improvement (QI) at a university-affiliated hospital.

**Results:**

Managers described that QI and lean shared the same overall purpose: focus on patient needs and improve efficiency and effectiveness. Employee involvement was emphasized in both strategies, as well as the support offered by managers of staff initiatives. QI was perceived as a strategy that could support structural changes at the organizational level whereas lean was seen as applicable at the operational level. Moreover, lean carried a negative connotation, lacked the credibility of QI, and was perceived as a management fad.

**Conclusions:**

Aspects of QI and lean were misunderstood. In a context where lean remains an abstract term, and staff associate lean with automotive applications and cost reduction, it may be fruitful for managers to invest time and resources to develop a strategy for continual improvement and utilize vocabulary that resonates with health care staff. This could reduce the risk that improvement efforts are rejected out of hand.

**Electronic supplementary material:**

The online version of this article (doi:10.1186/s12913-016-1838-z) contains supplementary material, which is available to authorized users.

## Background

Beginning in the 1980s, health care has experimented with many different quality improvement (QI) approaches [1–4]. The different approaches wash in and out in ebbs and flows lasting 3–5 years [4]. Applications are often superficial and key principles are often missed, misunderstood, or misused [5, 6]. The practical differences between the approaches have been more about terminology than substance. Hence, the pattern has been dubbed “pseudoinnovation”—the “repeated presentation of an essentially similar set of QI ideas and methods under different names and terminologies” [4].

That this pattern persists may be a result of the fact that the messengers and supporters of the new approaches have vested interests in writing books, articles, selling (consulting) services, or presenting at conferences [4]. It may also be that policy makers and health care organizations themselves desire and look for quick and simple “silver bullets” despite the evidence that points at the need for focus, discipline, patience, and hard work to solve complex problems [7].

### Quality improvement

Quality improvement can be seen as a relationship between people, process, and possibility. *People* are the motor that drives the work—the stakeholders and actors that must convene for something to happen. *Process* refers to the (scientific) approach to learning about and improving the organization, which leads to the *possibility* of improving quality, often defined in relation to the customer. In health care, quality improvement has been described as “…the combined and unceasing efforts of everyone—health care professionals, patients and their families, researchers, payers, planners and educators—to make the changes that will lead to better patient outcomes (health), better system performance (care) and better professional development” [8].

Due to the variations in success of QI efforts in health care, various strategies have developed to improve outcomes. These include the development of checklists [9] based on the identification of important facilitating factors, such as: support from top management, organizational culture and years of organizational involvement, physician involvement, IT infrastructure, and QI support [10]. The “Model of Improvement” prefaces the Shewhart Plan-Do-Study-Act (PDSA) improvement cycle with three questions: 1. What are we trying to accomplish?, 2. How will we know that a change is an improvement?, and 3. What change can we make that will result in an improvement? [11]. The Breakthrough Series, a collaborative model for achieving “breakthrough improvement” was developed at the Institute for Healthcare Improvement [12]. The idea was to bring together different providers who, through facilitated collaboration, can become faster at learning about and improving a specific topic area [13]. This approach is widely used in health care [14] and has informed many QI efforts at both local and national levels in Sweden, albeit with mixed results [15–19].

### Lean

The first empirical applications of lean in health care began in the mid-1990s, but it was not until 2002 that lean began to spread more widely [20]. In 2011, nine out of ten hospitals in Sweden claimed to have implemented lean at some level of the organization [21]. Historically, lean can be seen as the result of QI applications in the context of a post-World War II Japanese manufacturer (Toyota) that merged with that company’s history of innovation that began at the conclusion of the 19^th^ century. Toyota has since consistently worked to further their QI approach through iterative sustaining innovations. Eventually, Toyota’s economic success piqued the interest of a group of researchers in the late 1980’s who set about to understand what it was that they did differently [22]. The term “lean” was coined to describe Toyota’s replacement of the redundancy and robustness associated with working in batches with a process of pull based on the principles of just-in-time and automated quality. Lean can be defined as “a management practice based on the philosophy of continuously improving processes by either increasing customer value or reducing non-value adding activities (muda), process variation (mura), and poor work conditions (muri)” [23].

Applications of lean in health care have varied in the degree of fidelity they share with Toyota’s production system (TPS). In particular, divergence from TPS has been seen in the definition of customers and how customer value is generated as well as viewing lean as a tool-based approach instead of a philosophy or strategy [24]. Evidence on lean applications in health care show variations in the results obtained, partly due to the implementation strategies employed, support from top management, collaboration across organizational boundaries, and ability to engage multidisciplinary teams and invest in organizational support [25]. The complexity of the context in which lean is applied is also of vital importance [26].

### Understanding lean health care in the context of QI

Quality improvement and lean share a number of similarities (See Table 1), especially in terms of the underlying theories of improvement.

**Table 1:** Key similarities and differences between QI and Lean (inspired by [30])

Aspect	Lean	QI
Definition in health care	Continual improvement of processes by increasing value-adding activities and reducing non-value adding activities, variation, and bad work conditions [23].	Everyone’s continuous effort to create changes, that lead to better patient health, better care, and professional development [8].
Origins	Toyota’s production system and approach to quality improvement can be traced back to the company’s development of an automatic stopping device for looms in 1896. Among important contributors to this development have been the quality improvement thinking of Shewhart, Deming, and Juran in the 1950s [22, 30, 42–44].	Roots lie in physics, engineering, and manufacturing and the development of statistical methods and scientific approaches to understand and measure variation and thereby identify and eliminate sources of poor quality. By continually monitoring for and improving quality, a distinction was made in 1924 between quality assurance and quality improvement with the help of control charts. Key figures include Shewhart, Deming, and Juran, among others [30].
Applications in health care	The first empirical applications of lean in health care began in the mid-1990s, but it was not until 2002 that applications began to spread [20].	Since the late 1980s, health care has experimented with series of different quality improvement approaches [4, 45, 46].
Key principles	Jidoka (“automation with a human touch”, i.e. quality is built into the process) and Just-in-time (“only what is needed, when it is needed, and in the amount needed”) [44].	The “model of improvement” which links the Plan-Do-Study-Act (PDSA) cycle of iterative learning with three questions related to goal, measurement, and what to change [11].
Results	Factors related to lean success: comprehensive project organization, employee and safety-staff representation, and top management attendance [35].	Factors related to QI success: leadership from top management, QI experience, organizational culture, and data infrastructure/information systems [10].

In practice, QI has a longer history of applications in health care and there are differences in the specific principles and tools that are applied. Since the focus of lean applications in health care have primarily been at a practical tools-based level [25], one could surmise that practitioners perceive QI and lean as different. This could create confusion and trigger responses that influence the implementation of improvement efforts. How managers perceive the similarities and differences between the QI and lean improvement strategies may contribute to the phenomenon of management pseudoinnovation. The aim of this study was therefore to explore how hospital managers perceive lean in the context of quality improvement.

## Methods

### Study design

We used a qualitative approach with semi-structured interviews.

### Study setting

The study was conducted at a university affiliated tertiary-care Swedish hospital. The hospital has a focus on treating general population maladies. It serves a population of about 500,000 and has one of Europe’s largest emergency departments. In 2003, the hospital began to engage in systematic and continuous QI efforts. In 2005, the hospital participated in the first of several Swedish Breakthrough Series collaborations. The approach followed the guidelines described by IHI [13] and involved a start-up meeting with improvement teams from different health care organizations across Sweden faced with similar challenges related to a particular theme, such as emergency department waiting times. The groups were taught the basics of QI by facilitators from the Swedish Association of Local Authorities and Regions (SALAR) who then organized meetings at spaced intervals so that participants could learn from each other. The work was documented by each improvement team and summarized in reports published by SALAR [19].

In 2008, lean was introduced. At the same time, the hospital worked to capture their organizational values, first officially published in 2009. In it, continuous improvement was described as a core strategy that engaged employees throughout the entire organization, and lean was introduced as one of many possible methods to ensure this. The hospital invested in a lean training program for all employees. Lean efforts were confined to single units. Since 2011, the hospital has worked to translate organizational goals to the unit and employee levels and to adopt the Balanced Scorecard.

### Participants

At the behest of the CEO, we approached all members of the hospital’s top management team (CEO, chief medical officer, department chairs, and managers and directors of administrative and support services). Twelve members agreed to participate (ten were physicians, the other two had backgrounds in HR and finance; eight were women and four were men).

### Data collection

Interviews were conducted in Swedish by the third author, an experienced qualitative researcher with a background in psychology and management. Interviews were conducted at the participants’ workplace during working-hours between April and May, 2013, and lasted about one hour.

A semi-structured interview guide was created, pilot-tested, and refined (See Additional file 1). The focus was on perceptions of quality improvement and lean at the hospital and overall strategic issues. The questions were open-ended and allowed for follow-up questions to capture details and more in-depth information [27]. All interviews were digitally recorded and transcribed *verbatim*.

### Data analysis

Interview transcripts were analyzed using inductive content analysis [28]. All authors repeatedly read the interviews to gain familiarity with the data. We chose as our unit of analysis those sections in which lean or quality improvement were discussed. Meaning units were identified in these sections and condensed into codes. Codes were compared and divided into categories and sub-categories. This was done in parallel by the second and last authors to increase trustworthiness [29]. Finally, all categories were organized in themes that reflected the underlying (latent) meanings of the text by the first, second, and last author. Illustrative quotations were translated after analysis was completed and checked by a native English speaker fluent in Swedish. The data analysis was conducted in NVivo qualitative data analysis software; QSR International Pty Ltd. Version 10, 2012.

## Results

We identified 22 categories, which we grouped into nine themes. We present these in three groups and exemplify with quotations from different participants.

- 1. QI and lean share many similarities
  QI and lean were both described as strategies that made use of employees’ desire to create an effective and efficient organization. The majority of participants felt that both QI and lean led to a better working environment and lowered costs.
  - Theme 1: Purpose of QI and lean is to focus on patients and become effective and efficient
    Participants described that the purpose of QI and lean was to focus on patients—to develop a patient-centered approach with a high degree of patient safety. Another purpose was to become effective and efficient.
    …that you have the same good quality and the same access to care and maybe even increase your production, your health care production and accessibility, but with the same or fewer resources. (P12)
    Effectiveness was defined as the ability to identify quality deficiencies and to create value for the patient. Efficiency was related to the importance of avoiding duplication of work, finding and avoiding waste, and cost reduction.
  - Theme 2: QI and lean are change strategies to continuously improve care processes by engaging employees through the creation of a culture of improvement
    Participants described QI and lean as change strategies that contributed to the creation of a culture of improvement. Both promote continuous incremental change, with the intention of making work easier to perform correctly. Several participants mentioned that both QI and lean focused on care processes. They stressed the importance of minimizing the waiting time patients experience between tasks, and that care processes should make optimal use of resources, both personal and physical.
    Participants described the importance of engaging and actively involving employees in both QI and lean. Employee involvement was important because it led to commitment and continuity in the improvement process. It was described as a necessity that employees take responsibility for their own part in care delivery and for their contributions to the development of the organization. It was also important for both managers and employees to be self-critical:
    …this means having to look constantly at oneself and how one works, to be more critical; [this] is part of improvement, as I see it… (P12)
    Another way to engage employees was through the measurement and feedback of results. Participants described that it was important for employees themselves to measure and understand the reason for the measurements so that they will better understand the results.
  - Theme 3: Managers should be present and supportive and front-line staff should take initiatives
    For both QI and lean, participants stated that managers should be engaged and present. This included addressing employees by name and spending time on the floor to develop a better understanding of the work situation:
    …the managers should leave their nice rooms and actually get greater insight into the activities. (P5)
    Managers should support suggestions from front-line staff. Staff should take the initiative and generate their own proposals. Participants described the importance of authority mandated from above so that both managers and front-line staff could have the power and ability to influence.
  - Theme 4: QI and lean will result in a better work environment and lowered costs
    Participants described that QI and lean result in a better work environment due to a more structured way of working that was in line with hospital values. The participants also expressed the expectation that QI and lean interventions could lead to cost reductions.
    If we take the main focus, it is on economics, but employee and work environment values are a part of this too. (P11)
- What is QI?
  The main difference between QI and lean was that QI was described as a clear and comprehensible strategy to guide organization-wide change. Lean was described as vague, most applicable at the unit level, but generally of dubious relevance for health care.
  - Theme 5: QI is a natural fit for health care and an effective organizational strategy
    All participants described that QI was a natural fit for health care. Considerable doubt regarding the relevance of lean was expressed. Several mentioned the hospital’s effort to capture its values. The values described in the policy document were described as an important management tool. Participants explained that QI had emerged as a core value and this realization created a common foundation across units and among employees for continued engagement in QI.
    Participants described that QI led to changes in organizational structure, while lean was seen as specific to improvement in everyday work.
    …lean may be a way of working… to make both small and the large things more effective, but it is not a way with which to work with structural changes (P12)
  - Theme 6: QI enables staff and managers to question and improve work practices
    QI helped staff and managers to question how work was carried out. QI was associated with a positive “dissatisfaction” with current routine; to constantly be curious and interested in developing new ways of working. Participants described QI as daring to be self-critical and not worrying about prestige:
    To dare – dare to vet oneself, to be without prestige, and to see that there are always opportunities to do things in a different way, and to, somehow, dare to have that attitude… (P12)
    Participants described QI as a way to improve how things were done, develop smarter ways of working, discover smarter ways by setting SMART goals, and learn through PDSA cycles. QI helps the organization face the challenges associated with a constantly changing external environment:
    …the world is changing all the time, so what I mean is that it [QI] is not just about always developing new ways of working or new techniques, that is not even possible, but it is about being in step with [the environment] and maintaining a *takt* in the organization so that it adapts itself in the right way. That, well, that is the challenge. (P10)
    Participants stressed the importance of developing seamless care chains and providing better services. Participants also described that distance to patient care determines the focus of QI: Managers should focus on structural or organizational improvement and staff, who are closer to patients, should utilize their unique and practical knowledge and apply QI on operational improvements. It is important that the managers recognize and utilize the knowledge of staff on the floor. It is also vital that managers help each other to move beyond silo thinking. They need to develop an understanding of the bigger picture, a “helicopter perspective”:
    …that managers actually really help each other, that in our complex organization they leave their silos and see that, okay, the red numbers here probably depend on this, or maybe also that; that we must help each other and have a helicopter perspective and actually look horizontally… (P5)
    Participants recognized the existence of economic aspects in QI. They saw economic thinking as a base to build upon, which they contrasted with lean where they saw cost reduction as the ultimate goal.
- What is lean?
  - Theme 7: Lean is an operational strategy to improve care processes at the unit level
    Participants described lean as an operational strategy and QI as an organizational strategy. They felt that basic organizational principles such as how the hospital is structured can not be changed with lean. They thought these major structural changes were too difficult for lean because these were issues where it was difficult to achieve consensus, and they saw consensus as part of lean.
    Participants mentioned a number of operational sub-strategies connected to lean. These included working at the unit level and the importance of having structure and clear governance. Together, these lead to the development of organized work processes with clear task divisions for staff. To this end, it was important that the small lean improvement groups had the mandate to make their own decisions:
    …we have systematic improvement going on all around us, in small groups, each with its own mandate to make decisions. (P9)
  - Theme 8: Exercise caution with terminology
    Several of the participants mentioned that lean, in contrast to QI, had acquired a negative connotation throughout the organization. The concept had sometimes been used in an arrogant manner. They described that staff and managers did not always understand what to do with lean, nor what it amounted to. Some participants felt that lean had become something abstract, something people talked about without understanding it, and that staff were influenced by these negative opinions. Lean was often associated with Toyota and participants described that this had increased the skepticism of staff and managers towards the concept. This skepticism was compounded by what managers felt was a hidden economic agenda. A skepticism they felt staff shared.
    Yeah, they say that there are no economic agendas, but many do make that connection. And, many say that it is hidden, that when one talks about lean, what it *really* is about is to make things cheaper, and so forth… (P4)
    Participants described that some had written off lean as another fad that would soon disappear. All of this led some participants to avoid the term and instead discuss “flow”, “value-adding time”, or “value-creation”.
  - Theme 9: Lean is a philosophy
    Some participants differentiated QI and lean by describing lean as a philosophy. This philosophy manifested itself in a toolbox of methods and a number of sub-strategies, such as continuous improvement, the development of long-term goals, employee participation, the importance of leadership, and the development of a sustainable improvement culture. Lean was also described as an ideology based on common sense with sound principles.
    Lean – it is both a deep philosophical reasoning around continual improvement, it is somehow this entire culture… a long-term perspective, linked to an entire toolbox. (P9)

## Discussion

Hospital management team members described QI and lean as strategies to continually improve care processes that aim to meet patient needs while improving efficiency and the working environment. While they acknowledged that there are several similarities between QI and lean in terms of purpose, definition, roles of staff and managers, and the expected effects, they also differentiated between the two (Table 2).

**Table 2:** Comparison of participants’ understanding of lean and quality improvement

Aspect	Lean	QI
Purpose	To focus on patients and become effective and efficient (Theme 1)	
Definition	Change strategies to continuously improve care processes by engaging employees through the creation of a culture of improvement (Theme 2)	
Roles	Managers should be present and supportive; front-line staff should take initiatives (Theme 3)	
Expected effect	A better work environment and lowered costs (Theme 4)	
In practice	Lean is an operational strategy to improve care processes at the unit level (Theme 7) Lean is a philosophy incorporating strategies and a methods toolbox (Theme 9)	QI is a natural fit for health care and an effective organizational strategy (Theme 5)
Lessons learned	Exercise caution with terminology which staff might find foreign to health care (Theme 8)	QI enables staff and managers to question and improve work practices (Theme 6)

In practice, QI was described as a strategy that supports structural changes at the organizational level. Lean was described as a method applicable at the operational level to improve processes within single units. In terms of the lessons that managers had learned, lean was, in comparison to QI, perceived to be more controversial among staff. Participants suggested that this may be due to the strong association lean has among staff to manufacturing and a “hidden economic agenda”. The credibility of lean may be further hampered due to its perception among staff as another management fad. While both QI and lean can trace their roots to manufacturing and engineering, it was the relevance of lean that was questioned. In Sweden, lean was introduced concurrently with a national drive to cut waiting times and improve access to care. Yet, participants associated decreased waiting times with both QI and lean.

That managers perceive QI and lean as strategies to continually improve care processes is not surprising and is consistent with how QI and lean historically developed in parallel. The similarities between QI and lean identified in this study are consistent with those emphasized in a recent report by the IHI [30]. However, the fact that top managers did not see or understand lean as an organization-wide improvement approach may reflect the inability of many health care organizations to fully implement lean [25, 26]. Turning to the literature, we find that lean entails an end-to-end approach to process improvement that crosses organizational boundaries [31, 32]. Indeed, it is due to this very focus on the development of end-to-end value streams that lean has been described as a strategy integral to the achievement of the triple aim [33]. Lean implies the replacement of a functional organization with a process-based organization [34], i.e. lean has organizational and structural implications. When health care organizations manage to implement lean across organizational boundaries, the gains can be large and results can be sustained in the long term [35]. However, the shift towards a process-oriented organization in health care has proven difficult in Sweden and internationally [25, 26, 36]. It may be possible to attribute this difficulty to the interpretation, as illustrated by the participants’, that lean has primarily an operational focus consisting of a methods toolbox and is not concerned with larger structural changes at the organizational level.

Managers interpreted the negative associations of staff to lean as a feeling that it was just another fad and often linked it to cost-cutting measures. It is remarkable that QI did not carry the same associations. The view on cost presents an interesting paradox: hospital managers recognized the need to cut costs and saw QI and lean as strategies to achieve cost savings. At the same time, they described that staff were wary of lean because they suspected there was a hidden agenda to cut costs and that lean was the means to fulfill this agenda. Both QI and lean are based on the idea that improvements in quality lead to cost reductions because there is less need to spend resources to fix things that have been done incorrectly [37]. That a misunderstanding persists may be due to the lack of costing models that clearly demonstrate a causality between quality improvements and cost savings [38]. If quality improvement approaches continue to be implemented with a limited understanding of their core principles [5, 6], implementation risks becoming superficial as well. This may be counteracted through a clear strategic direction aligned with continuous investments in QI, which has been shown to be effective in sustaining improvement in health care [7].

The manufacturing roots of lean were described as a challenge to implementation. This is corroborated by the literature [39, 40]. It could be that lean was exemplified with links to Toyota and car manufacturing from the start. However, a similar association was not made with QI, which also has its roots in manufacturing. Perhaps health care is not as aware of the history of QI, or perhaps it is a function of time. The literature seems to support this—eventually staff can become familiar with a QI idea, even if the full implications have not been understood [4, 6]. Over time, QI may have become a concept, dissociated from its tools, in comparison to lean.

Toyota did not implement lean nor directly copy QI as we are wont to do in health care. Instead, they let their own long-term strategy and culture of improvement evolve. Those organizations most able to develop and sustain organization-wide QI and lean initiatives such as Intermountain Healthcare and Virginia Mason, respectively, actually avoided the terms and instead focused on the development of their own production or care systems in a way that was consistent with their organizational values [25, 41]. It appears to be important to develop and link organizational culture with QI and lean through a focus on meaningful principles, such as patient needs [30]. Indeed, the organization in question has recently abandoned the term lean and focuses now on their core set of values to guide improvement efforts.

### Methodological considerations

In the organization studied, QI was used as the overall improvement strategy since 2003 and lean was introduced in 2008. At the time of the interviews, participants’ understanding was relative to the differences in organizational experience (QI for 10 years, lean for five). At the national level, Sweden has seen widespread applications of lean. In 2011, 90 % of hospitals in Sweden claimed to be working with lean [21]. This has led to considerable public debate, which might have contributed to the negative connotations associated with lean.

## Conclusions

While QI and lean share some of the same core principles and theories, this study suggests that due to the negative connotations associated with lean, care should be used in selecting those strategies and terminologies that resonate with health care staff and managers. However, in the end, it may make no difference if QI is seen as an organizational strategy and lean as a toolbox. What does make the difference is if an organization lets the understanding of its members regarding a specific improvement strategy such as lean muddy the waters. Then the organization runs the risk of developing organizational myopia and losing sight of the larger picture and purpose because it limits itself to unit-based and silo-focused improvements that do not challenge organizational boundaries. Given the high degree of similarity among different QI approaches, what is perhaps most important is that organizations develop their own long-term and systematic approach to the evolution of improvement, one rooted in the values and culture of the organization and shared by its members.

## Additional file

Additional file 1: Interview Guide. Description of data: English translation of the interview guide used in the study. The original Swedish language version of the interview guide is available upon request from the authors. (DOCX 31 kb)
