# Supplementary material for: Does lean muddy the quality improvement waters? A qualitative study of how a hospital management team understands lean in the context of quality improvement
Source: BMC Health Serv Res. 2016 Oct 19;16:588. doi: 10.1186/s12913-016-1838-z (PMC5069852; doi:10.1186/s12913-016-1838-z)
Supplement: Additional file 1: — Interview Guide. Description of data: English translation of the interview guide used in the study. The original Swedish language version of the interview guide is available upon request from the authors. (DOCX 31 kb) [file 12913_2016_1838_MOESM1_ESM.docx]

Supplementary File 1 - Interview Guide

# Introduction

The purpose of this interview with the members of the hospital management team is to try to understand more about the leadership of improvement efforts and the role of managers in those efforts, as well as to better understand the current situation at the hospital. The interview will be digitally recorded. I will also take notes during the interview in case the recording malfunctions.

Is this ok with you?

The interview will take one hour. I will ask questions using an interview guide focused on different areas. Everything that will be said during the interview will be treated with care and will only be viewed by the research group. The results will be presented anonymously and aggregated at the group level. A preliminary analysis will be presented for the management group. The interviews will also be used for research about the leadership of improvement. Your participation is voluntary and you can decided to discontinue your participation at any time.

If this is ok with you, perhaps we can start?

# Opening Questions

1. You are a member of the management team at this hospital. Could you briefly describe your role(s) and your tasks in the organization and which areas you are responsible for?
2. What does improvement mean to you? What do you include in the term?

# Main Questions

1. How would you describe the hospital’s approach to improvement today?
2. How would you describe the approach to improvement you have in the areas you are responsible for?
3. How do you see the role you have in this work?
4. How do you understand and view the planned efforts to improve the hospital and your role as a manager in that work?
5. What do you believe is required of you and your middle managers to lead these improvement efforts in an effective way?
6. What do you believe is required of your CEO to be able to lead improvement efforts in an effective way?
7. When you hear the word “lean”, what do you think about then?
8. How have you developed your general competencies (knowledge, skills, attitudes) as a manager and leader?
9. How have you developed your competencies (knowledge, skills, attitudes) for leading improvement efforts?
10. How have you developed your competencies (knowledge, skills, attitudes) for leading lean efforts?

# Concluding Questions

1. What would you emphasize as the most important thing regarding the development of effective leadership of improvement efforts within this hospital and within health care?
2. Is there anything that you would like to add regarding the leadership of improvement efforts or anything else that we have discussed?
